# Highly stretchable and robust transparent conductive polymer composites for multifunctional healthcare monitoring

**DOI:** 10.1080/14686996.2022.2070864

**Published:** 2022-05-24

**Authors:** Anky Fitrian Wibowo, Joo Won Han, Jung Ha Kim, Ajeng Prameswati, Siti Aisyah Nurmaulia Entifar, Jihyun Park, Jonghee Lee, Soyeon Kim, Dong Chan Lim, Myoung-Woon Moon, Min-Seok Kim, Yong Hyun Kim

**Affiliations:** a Department of Smart Green Technology Engineering, Pukyong National University, Busan, Republic of Korea; b Industry-University Cooperation Foundation, Pukyong National University, Busan, Republic of Korea; c Department of Creative Convergence Engineering, Hanbat National University, Daejon, Republic of Korea; d Surface Technology Division, Korea Institute of Materials Science (KIMS), Changwon, Republic of Korea; e Department of Materials and Life Science Research Division, Korea Institute of Science and Technology, Seoul, Republic of Korea

**Keywords:** Natural rubbers, PEDOT:PSS, stretchable electronics, stretchable sensors, wearable electronics

## Abstract

Soft, stretchable, conductive thin films have propelled to the forefront of applications in stretchable sensors for on-skin health monitoring. Stretchable conductive films require high conformability, stretchability, and mechanical/chemical stability when integrated into the skin. Here, we present a highly stretchable, conductive, and transparent natural rubber/silver nanowire (AgNW)/poly(3,4-ethylenedioxythiophene):poly(styrenesulfonate) (PEDOT:PSS) composite film. Overcoating the PEDOT:PSS layer results in outstanding mechanical robustness and chemical stability by suppressing the mechanical and chemical degradation of the nanowire networks. Moreover, the introduction of the organic surface modifier enhances the bonding strength between the natural rubber substrate and AgNW at the interface. The highly conformable composite films are integrated into multifunctional on-skin sensors for monitoring various human motions and biological signals with low-power consumption. We believe that the highly stretchable, robust, and conformable natural rubber/AgNW/PEDOT:PSS composite film can offer new opportunities for next-generation wearable sensors for body motion and physiological monitoring.

## Graphical abstract

**Figure:**
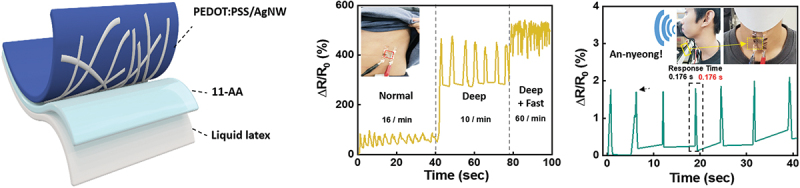

## 1. Introduction

On-skin sensors have attained a place of prominence in healthcare monitoring because of their capability to sense various motions and biological signals in the human body [1–4]. To realize high-performance skin-attachable sensors, it is necessary to develop efficient substrate materials which can provide high conformability and stretchability. Advances in on-skin sensors have been accompanied by extensive research on stretchable electronic devices with high stretchability, softness, conformability, and sensitivity [5–7]. More importantly, reducing physical mismatches between the sensor substrates and human skin is important for the sensors to conform and integrate with contours of the human body. Polydimethylsiloxane (PDMS) and polyurethane are the most widely used substrates in stretchable electronics because of their easy processability [8–10]. However, the high material cost of PDMS and the poor weatherability of polyurethane limit their practical applications in on-skin electronics. Natural rubber is a promising candidate for stretchable transparent substrates of skin-attachable sensors owing to its outstanding stretchability, conformability, eco-friendliness, easy processability, and low material cost. These properties enable the realization of highly soft and wearable skin-attachable sensors, allowing them to conform to human skin [11,12].

Stretchable transparent electrodes are also of great importance in optoelectronic devices that require high stretchability [13–15]. Owing to their superior electrical conductivity, mechanical flexibility, processability, optical transmittance, and low cost, silver nanowires (AgNWs) have attracted tremendous interest as electrode materials for optoelectronic devices and wearable electronic sensors to replace conventional indium tin oxide (ITO) transparent electrodes [16,17]. Although ITO is the most commonly used transparent electrode, its inherent brittleness, high material costs, and high processing temperature restrict its application in low-cost, flexible, and stretchable electronic devices. Recently, stretchable AgNWs have shown great potential for various applications including soft robotics sensors [18,19], stretchable transparent heaters [20], electromagnetic interference shielding [21], supercapacitors [22,23], touch panels, and conductors [24]. Copper nanowires are also attracting considerable attention as stretchable transparent electrodes for flexible and stretchable electronics due to their low cost, reliable mechanical, thermal, and electrical properties [25,26]. Since the conductivity of nanowire-based films is dominated by wire-to-wire junction resistances, various welding processes have been made to reduce the junction resistance of nanowires, including thermal, electron beam, joule heating, intense pulsed light annealing and etc [27–30]. Lee et al. reported the polymer-assisted soldering technique for tightening the junction of AgNW networks which reduces the contact resistance and achieves a high transmittance with high mechanical stability [31]. Despite the advantages of AgNWs, it is challenging to use nanowires in stretchable applications because of their poor adhesion to elastomeric substrates which are strongly hydrophobic [32–35]. Although various surface functionalization approaches for elastomeric substrates have been reported, limited interface adhesion is still a challenge [33–39].

Here, we present robust, stretchable, conductive, transparent, and conformable natural rubber/AgNW/poly(3,4-ethylenedioxythiophene):poly(styrenesulfonate) (PEDOT:PSS) composite films. An organic surface modifier, 11-aminoundecanoic acid (11-AA), remarkably enhanced the bonding strength at the interface between the natural rubber substrate and AgNW. In addition, the electrical and mechanical properties of composite films were significantly improved by introducing a fluorosurfactant to the PEDOT:PSS film, which enhanced the wettability of PEDOT:PSS on the natural rubber/AgNW film. Owing to the suppression of the mechanical and chemical degradation of nanowire networks by PEDOT:PSS overcoating, the composite films with the PEDOT:PSS layer resulted in enhanced mechanical robustness and chemical stability compared to the film without the PEDOT:PSS layer. Based on the optimized composite films, we successfully developed a high-performance multifunctional on-skin sensor for monitoring the human body. The sensors yield outstanding sensing performance with low-power consumption for large body motions at the wrist, palm, finger, knee, and sole of the foot and conform closely to body contours. Moreover, the sensors enable the monitoring of subtle vibrational forces in the form of speaking, breathing, and blinking eyes. We believe that the highly stretchable, robust, and conformable natural rubber/AgNW/PEDOT:PSS composite films can be a key step toward skin-attachable wearable sensors for monitoring physical activity as well as medical and physiological signals.

## 2. Experimental section

Materials: Liquid natural rubber was purchased from SFX Korea. Poly(3,4-ethylenedioxythiophene):poly(styrene sulfonate) (PEDOT:PSS, Clevios PH1000) was purchased from Heraeus. Silver nanowire (AgNW, diameter: ~21 nm, length: ~22 μm) suspension was purchased from Flexio Co., Ltd. The 11-aminoundecanoicacid (11-AA) powder was procured from Sigma Aldrich.

Preparation of natural rubber substrate: The fabrication of natural rubber substrates was performed in a simple manner using the spin-coating method. Natural rubber solution in the liquid state, having a concentration of 1 ml was spin-coated on glass substrates at a spin speed of 500 rpm for 10 s and subsequently, annealed on a hot plate at a temperature of 100°C for 5 min. This process was repeated several times to achieve optimal film thickness.

Preparation of 11-aminoundecanoic acid solution: 4 mg of 11-AA powder was diluted with 2 ml of deionised water. The solution was stirred on a hot plate at 1000 rpm and 60°C for 1 h. Then, 8 ml of anhydrous ethyl alcohol was added to the solution and sonicated for 1 h to homogenise the solutions. Subsequently, the solution was heated on a hot plate at 1000 rpm and 60°C for 1 d.

Fabrication of natural rubber/AgNW/PEDOT:PSS composite films: The fabrication procedure for the natural rubber/AgNW/PEDOT:PSS composite films is schematically illustrated in Figure 2. The natural rubber substrate was pre-treated with oxygen plasma for 30 min to obtain hydrophilicity. The 11-AA surface modifier was spin-coated onto the plasma-treated natural rubber substrate at a spin speed of 500 rpm for 30 s and subsequently annealed at 100°C to generate a strong bond between the AgNW networks and the natural rubber substrate. The AgNW suspension was spin-coated on the natural rubber substrate and annealed at 100°C for 10 min. This process was repeated thrice to produce three-layer AgNW films with high conductivity. PEDOT:PSS solution (10 ml) mixed with 6 ml of ethylene glycol and 10 µL of FS-31 was spin-coated onto the natural rubber/AgNW film at 3000 rpm for 30 s and annealed at 120°C for 10 min.

Characterization: The sheet resistance of the films was measured by the van der Pauw method using a source-meter unit instrument (Keithley 2401). The transmittance was measured using a UV-Vis spectrophotometer (UV-2600). The film thickness was measured by a surface profilometer (DektakXT, BRUKER). To detect the strain-resistance, copper tapes were attached to both ends of the composite films and wired to a source-meter unit instrument (Keithley 2401) to form a strain sensor. To monitor human movements, multi-functional sensors were attached to the human body, and the change in resistance signals was directly recorded using different human motions.

## 3. Results and discussion

**Figure 1.**
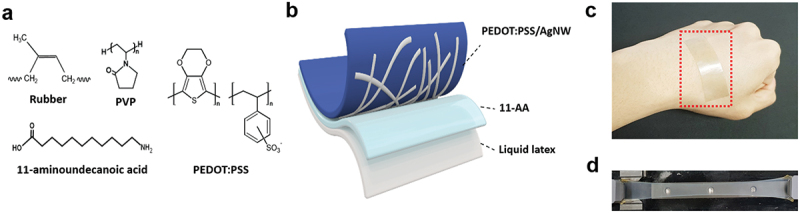
(a) Chemical structures of natural rubber, PVP, 11-AA, and PEDOT:PSS. (b) Schematic of the natural rubber/AgNW/PEDOT:PSS composite film. (c) Photograph of the composite film attached on skin. (d) Photograph of the composite film stretched up to 1350%.

Figure 1(a) shows the chemical structures of the materials used to prepare the composite films. A schematic of the film layout is shown in Figure 1(b). A surface modifier, 11-aminoundecanoic acid (11-AA), was introduced to enhance the adhesion between conductive AgNW networks and highly hydrophobic natural rubber substrates. The composite film can be conformally attached to human skin owing to its high elasticity and softness. (Figure 1(c)). Furthermore, the natural rubber substrate for composites exhibited a high transmittance of approximately 80% (at 550 nm), outstanding thermal stability (stable up to 250°C), and excellent stretchability. The composite film is capable of being stretched up to 1350% without tearing (Figure 1(d)).

**Figure 2.**
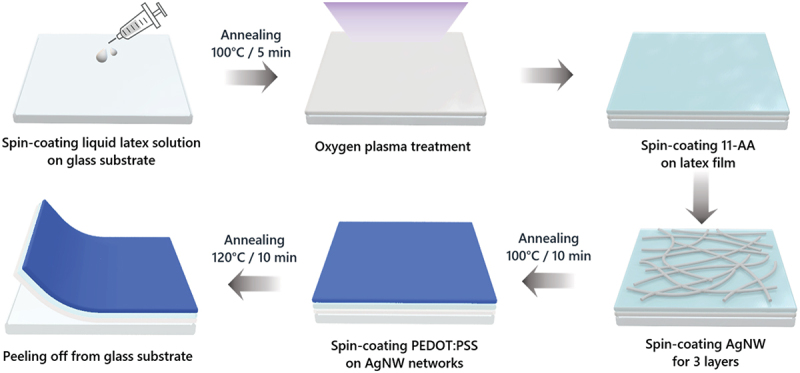
Schematic of the procedure for fabricating the natural rubber/AgNW/PEDOT:PSS composite film.

A schematic of the fabrication procedure for the natural rubber/AgNW/PEDOT:PSS composite film is depicted in Figure 2. Natural rubber solution in the liquid state was spin-coated onto the glass substrate and subsequently annealed. The natural rubber substrate was treated with oxygen plasma to create –OH functional groups on the surface. The primary amine groups (–NH_2_) and –OH groups of 11-AA are coupled with the low surface energy nonpolar regions of the natural rubber surface. Surface functionalization through 11-AA treatment increases the surface energy of natural rubber films; thus, the AgNW networks could be uniformly deposited on the surface. Additionally, primary amines (-NH_2_) of 11-AA form hydrogen bonds with polyvinylpyrrolidone (PVP) surrounding the nanowires, leading to a significant improvement in the bonding strength between the natural rubber substrates and nanowires. After the surface treatment, three-layered AgNWs were deposited on the film and then cured. Subsequently, the AgNW networks were embedded with a highly conductive PEDOT:PSS layer. The PEDOT:PSS solution mixed with non-ionic fluorosurfactant (FS-31) and ethylene glycol was spin-coated onto the AgNW-coated film to enhance the electrical and mechanical properties as well as chemical stability of the film. After drying, the composite film was peeled off the glass substrate.

**Figure 3.**
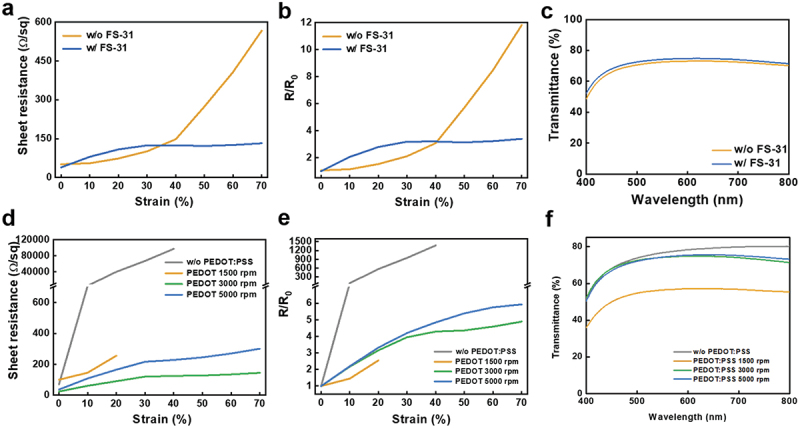
(a) Sheet resistance and (b) relative resistance changes under various strains for natural rubber/AgNW/PEDOT:PSS (w/ and w/o FS-31) composite films. (c) Transmittance spectra of the composite films w/ and w/o FS-31 in PEDOT:PSS. (d) Sheet resistance and (e) relative resistance changes under various strains for natural rubber/AgNW/PEDOT:PSS composite films prepared with different spin-speeds of PEDOT:PSS. (f) Transmittance spectra of the composite films with PEDOT:PSS prepared with different spin-speeds.

Figure 3(a-c) show the effect of the FS-31 fluorosurfactant in the PEDOT:PSS solution on the electrical and optical properties of natural rubber/AgNW/PEDOT:PSS composite films. The composite films with and without FS-31 show initial sheet resistances of 38.9 and 48.0 ohm/sq, respectively. While the composite film without FS-31 exhibits an 11.4-fold (547.3 ohm/sq) increase in resistance at a strain of 70%, a remarkably lower change is observed for the FS-31-doped film which registers an increase of only 3.45 times (134.5 ohm/sq) when stretched up to 70% (Figure 3(a-b)). The improved electrical performance and stretchability of the films by introducing the FS-31 surfactant are attributed to the enhanced wettability of PEDOT:PSS on the natural rubber/AgNW. The composite film with FS-31 also shows a higher average transmittance of 71.9% (400–800 nm) compared to the untreated film (70.1%), as shown in Figure 3(c). It is expected that the fluorocarbon backbone of FS-31 with a terminal part of a hydroxyl group would improve the wettability of PEDOT:PSS on the hydrophobic film effectively, resulting in uniform film formation.

Next, we investigated the effect of the PEDOT:PSS thickness on the properties of the composite films as a function of the tensile strain. Figure 3(d) shows the changes in the sheet resistance of the composite films under a strain of up to 70%. The film without the PEDOT:PSS layer shows an initial sheet resistance of 70.8 ohm/sq. The initial sheet resistances of the composite films integrated with the PEDOT:PSS layers prepared at spin speeds of 1500, 3000, and 5000 rpm were 100.4, 29.5, and 48.5, respectively, and their corresponding thicknesses for the PEDOT:PSS layers were 79, 42, and 23 nm, respectively. The lowest sheet resistance was observed for the film with PEDOT:PSS prepared at 3000 rpm. It is interesting to note that the film with the thickest PEDOT:PSS layer (79 nm, 1500 rpm) yields the highest sheet resistance compared to other films because of the inhomogeneous coating of the PEDOT:PSS layer which cannot distribute the carriers uniformly over the films. When a tensile strain was applied to the composite films, the film without the PEDOT:PSS layer showed a rapid increase in resistance with increasing strain and loss of conductivity above a strain of 20%. Composite films with the PEDOT:PSS layer prepared at spin speeds of 3000 rpm and 5000 rpm showed a more stable and limited increase in resistance change (Figure 3(e)). The relative sheet resistance changes of the films when stretched up to 70% were 4.9- and 6.2-fold, respectively. This result indicates that the PEDOT:PSS overcoat layer effectively prevents the fracture or dissociation of nanowires and yields a uniform distribution of electrical pathways.

The transmittance of the composite films decreased with increasing thickness of the overcoated PEDOT:PSS layers (Figure 3(f)). The lowest average transmittance of 54.4% was observed for PEDOT:PSS prepared at a spin speed of 1500 rpm. The composite films with the PEDOT:PSS layers prepared at 3000 rpm and 5000 rpm showed slightly decreased transmittance values of 71.9 and 72.2%, respectively. The pure natural rubber substrate and the AgNW film on the natural rubber without the PEDOT:PSS overcoat layer exhibited transmittances of 79.3 and 75.6%, respectively. These results prove that the transmittance slightly degrades with an increase in the thickness of PEDOT:PSS. The transparent conductive film for sensor applications is beneficial for electrophysiological sensors, allowing parts of target surfaces such as human skin to be visually observed during measurement and facilitating clinical trials in overcoming obstacles during real-time sensing operations [39]. In addition, the conductive material with the high transmittance can be utilized as a transparent electrode for various optoelectronic devices. We chose a composite film with overcoated PEDOT:PSS at 3000 rpm for application in sensors owing to its low resistance and high transmittance.

**Figure 4.**
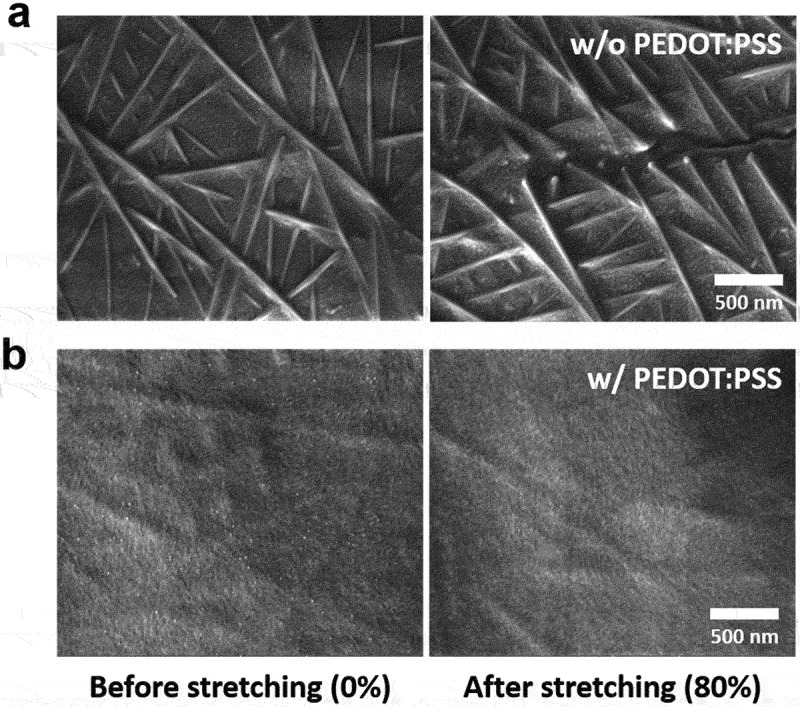
SEM images of (a) natural rubber/AgNW and (b) natural rubber/AgNW/PEDOT:PSS composite films under 0 and 80% tensile strains.

We observed scanning electron microscopy (SEM) images of the films to investigate the morphological effects of the PEDOT:PSS overcoat layer (Figure 4). The natural rubber/AgNW film clearly shows the fracture of the nanowires under an applied strain of 80%. In contrast, the composite film with PEDOT:PSS prevents the disconnection or fracture of nanowires at the same strain.

**Figure 5.**
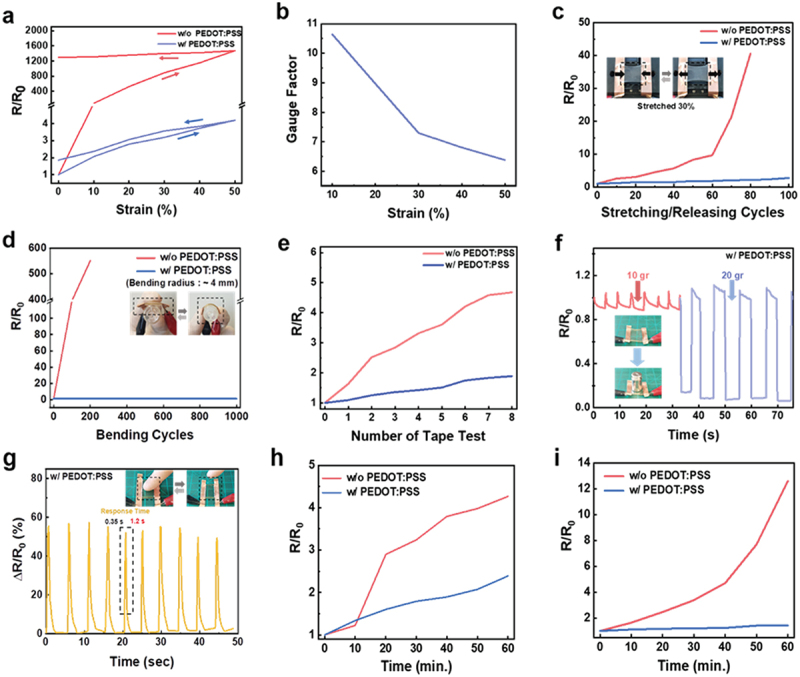
(a) Relative resistance changes of composite films with and without PEDOT:PSS layers as a function of tensile strains. (b) Gauge factor of the composite film with the PEDOT:PSS layer. Relative resistance changes of composite films with and without PEDOT:PSS layers as a function of (c) stretching-releasing cycles (strain: 30%), (d) bending cycles(bending radius: 4 mm), (e) the number of tape attach-detach test. Real-time relative resistance changes of the PEDOT:PSS-coated films induced by f)compressing free-standing films (weight: 10 and 20 gram) and (g) touching by finger. Relative resistance changes of composite films with and without PEDOT:PSS layers, which are dipped in (h) ethanol and (i) IPA.

Figure 5(a) displays the relative resistance changes of the natural rubber/AgNW films with and without PEDOT:PSS overcoat layers as a function of strain. The PEDOT:PSS-coated composite film shows suppressed hysteresis behaviour, while the natural rubber/AgNW film without the PEDOT:PSS layer presents a remarkably large hysteresis. The PEDOT:PSS-coated composite film achieves a high gauge factor of ~10.6 at a tensile strain of 10% (Figure 5(b)). We performed a cyclic stretch-release test for films under a strain of 30% (Figure 5(c)). With the help of the PEDOT:PSS overcoat layer, the composite films with PEDOT:PSS showed a limited change in resistance (2.76-fold) compared to the film without PEDOT:PSS during the repeated stretch-release cycles, which enables a low-power consumption and a low hysteresis of the wearable sensors. Moreover, the composite film with PEDOT:PSS retained its initial resistance even after 1000 cycles of bending tests with a small bending radius of ~4 mm, indicating high robustness, whereas the film without PEDOT:PSS exhibited a larger resistance change and lost its electrical property at only 200 bending cycles (Figure 5(d)). In addition, the PEDOT:PSS-coated film showed robust characteristics with a small resistance change over a few cycles of tape test (Figure 5(e)). The composite film with PEDOT:PSS successfully detects external stimuli such as compression and touching in real-time, showing clear resistance changes upon input load (Figure 5(f)) and touching the surface of film (Figure 5(g)). These results suggest that the PEDOT:PSS overcoat layer effectively disperses the applied stress across the entire composite film and provides outstanding mechanical robustness.

The composite films also exhibited robust chemical stability when the films were immersed in ethanol and isopropyl alcohol (IPA) (Figure 5(h-i)). Comparing the natural rubber/AgNW film without PEDOT:PSS, the relative resistance changes of the composite films with PEDOT:PSS are much smaller than those of ethanol, and almost negligible against IPA. The composite films with the PEDOT:PSS overcoat layer exhibited high mechanical robustness, chemical stability, and durability compared to the film without the PEDOT:PSS layer because overcoating the PEDOT..PSS layer enhances the durability of films by suppressing the deformation of nanowires under mechanical deformation and prevents chemicals from penetrating the film.

**Figure 6.**
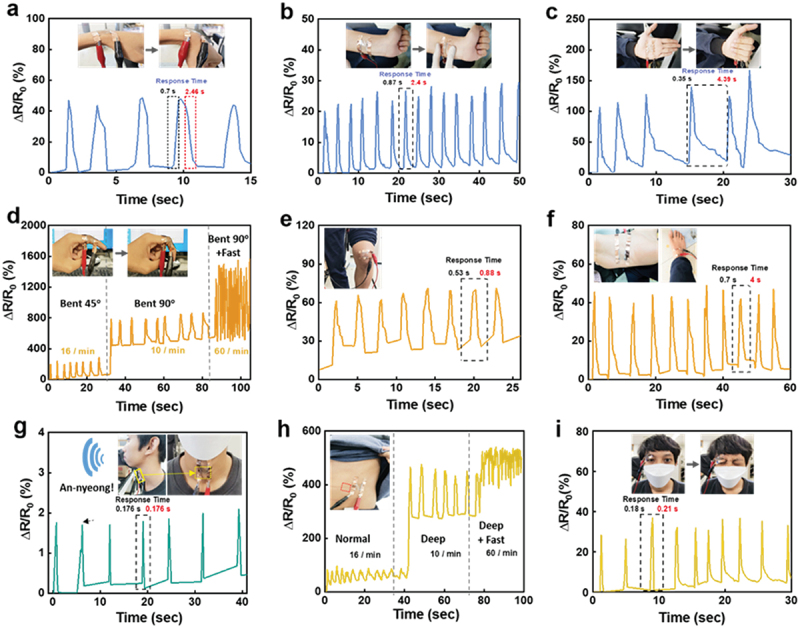
Detection of various human activities using on-skin sensors. Responsive curves of the sensors on (a, b) wrist, (c) palm, (d) finger, (e) knee, (f) sole of the foot, (g) voice, (h) breathing, and (i) blinking eyes.

To demonstrate the concept of on-skin strain sensors, we integrated natural rubber/AgNW/PEDOT:PSS composite films, which can respond to the changes in resistance, to the human body. Sensors with composite films enable the monitoring of various human activities, including joint or muscle movements of the wrist, palm, finger, knee, and sole of the foot. Figure 6(a) shows the relative resistance change (ΔR/R_0_) in natural rubber/AgNW/PEDOT:PSS composite films mounted on the wrist and repeatedly bent and straightened by approximately 90°. The change in the relative resistance shows a synchronous response with an average change of 45%. Figure 6(b) illustrates the relative changes in resistance with skin distortion caused by wrinkles. The sensors can conform to deformations along the skin. The relative resistance changes in a stable manner to an average of approximately 25% when the skin is wrinkled and repeatedly returns to its original state. Figure 6(c) shows the relative changes in the resistance of the sensor attached to the palm during the detection of extending/grasping motions. The grasping motion provides a greater distortion force compared to wrinkles on the skin, achieving a relative resistance change that exceeds 100%. In Figure 6(d), we observe that the sensor signal for finger bending is larger than that for wrist bending because of the higher strain associated with the bent finger. When the finger is bent gently at approximately 45°, the relative resistance change increases up to approximately 300%. When the finger is significantly bent to 90°, a larger change in the relative resistance up to approximately 800% may be observed. Moreover, the quick bending motion at 90° generated fast response signals. As the finger is straightened, the resistance returns to its original value in each bending state. The natural rubber/AgNW/PEDOT:PSS composite sensors can detect common foot movements such as walking and stepping on the floor (Figure 6(e-f)). The sensors were attached to the knee and the sole of the foot. The sensors detect the signal from a flexing/extending motion of the knee, while a ~50 kg person walks normally (Figure 6(e)). Relative resistance changes of approximately 60% were recorded during walking. The sensor attached to the sole of the foot exhibits a sensing signal with a relative resistance change of approximately 40% when a person stepped on the floor (Figure 6(f)). These results imply that the natural rubber/AgNW/PEDOT:PSS composite sensors effectively monitored the dynamic signals of human walking.

Our sensors can monitor more sensitive subtle motion signals, such as voice, breathing, and blinking movement (Figure 6(g-i)). The stretchable composite sensors were attached to the throat to detect different real-time quasi-periodic vibrations of the larynx produced by a phonation of ‘An-nyeong!’ (means “hello!’ in Korean), as shown in Figure 6(g). When the larynx experiences vibrations, the sensor detects the shrinkage deformation of the throat muscles, leading to a sharp change in resistance. The sensors exhibit remarkable sensitivity to larynx movements during phonation, indicating great potential for applications in speech rehabilitation training and in the medical field for detecting cough intensity. Furthermore, our sensors were capable of measuring the respiration process. The sensor was placed on the lower part of the chest and the relative resistance changes during breathing were monitored (Figure 6(h)). The expansion and contraction of the rib cage during the inhalation and exhalation processes induced relative resistance changes of the sensor by up to approximately 60% (normal breathing) and 400% (deep breathing). A fast response signal of approximately 500% was observed during deep and rapid breathing. These results demonstrate the capabilities of the strain sensor in recording subtle motions and distinguishing the degree of breathing which can monitor human physiological activities. In addition to the larynx and diaphragm movements, the sensors can promptly detect signals corresponding to tiny movements, such as blinking eyes (Figure 6(i)). The sensor attached to the eyelid successfully detected the relative resistance change generated by eye blinking. The relative resistance change of the sensor was increased by up to 35% by blinking the eye. Note that our sensors studied here exhibited a very low-power consumption in the range of a few microwatt. Our on-skin sensors based on natural rubber/AgNW/PEDOT:PSS composite films demonstrate diverse applications on the human body with high sensitivity, which has potential applications in wearable devices for medical and physiological treatments.

## 4. Conclusions

In summary, we demonstrate highly stretchable, conductive, and transparent natural rubber/AgNW/PEDOT:PSS composite films which show excellent conformability. The interface between the natural rubber substrate and the AgNW was functionalized by an 11-AA organic surface modifier which significantly improved the bonding strength at the interface. Furthermore, the introduction of the fluorosurfactant to the PEDOT:PSS film significantly enhanced the electrical and mechanical properties of the composite films by improving the wettability of PEDOT:PSS on the natural rubber/AgNW film. The composite films with the PEDOT:PSS layer yielded much higher mechanical robustness and chemical stability compared to the film without the PEDOT:PSS layer by suppressing the mechanical and chemical degradation of nanowire networks resulting from the PEDOT:PSS overcoating. The optimised composite films were used as multifunctional on-skin sensor for human body monitoring. The sensors conform closely to the contours of the human body and exhibit excellent sensing performance with low-power consumption for large body motions at the wrist, palm, finger, knee, and sole of the foot. Furthermore, subtle vibrations due to speaking, breathing, and blinking eyes could be monitored. These highly stretchable, robust, and conformable natural rubber/AgNW/PEDOT:PSS composite films are poised to contribute to the development of skin-attachable wearable sensors for monitoring physical activity as well as medical and physiological signals.

## Supplementary Material

Supplemental Material

## Supporting information

Transmittance of the films, SEM images of the composite films under various tensile strains, photograph of the 1350% stretching of the composite films, relative resistance changes of the films dipped in water, real-time sensors for monitoring repetitive head turning, summary of the mechanical properties in stretchable films, Sheet resistance and transmittance of the AgNW films, and detailed information of materials in the experiment.
