# Supplementary material for: Highly stretchable and robust transparent conductive polymer composites for multifunctional healthcare monitoring
Source: Sci Technol Adv Mater. 2022 May 24;23(1):332–40. doi: 10.1080/14686996.2022.2070864 (PMC9132465; doi:10.1080/14686996.2022.2070864)
Supplement: Supplemental Material [file TSTA_A_2070864_SM0416.doc]

**Supporting Information**

**Highly Stretchable and Robust Transparent Conductive Polymer Composites for Multifunctional Healthcare Monitoring**

Anky Fitrian Wibowo^a^, Joo Won Han^b^, Jung Ha Kim^a^, Ajeng Prameswati^a^, Siti Aisyah Nurmaulia Entifar^a^, Jihyun Park^a^, Jonghee Lee^c^, Soyeon Kim^d^, Dong Chan Lim^d^, Myoung-Woon Moon^e^, Min-Seok Kim^e,*^, and Yong Hyun Kim^a,*^

*^a^Department of Smart Green Technology Engineering, Pukyong National University, Busan 48513, Republic of Korea; ^b^Industry-University Cooperation Foundation, Pukyong National University, Busan 48513, Republic of Korea; ^c^Department of Creative Convergence Engineering, Hanbat National University, Daejon 34158, Republic of Korea; ^d^Surface Technology Division, Korea Institute of Materials Science (KIMS), Changwon-daero 797, Changwon 51508, Republic of Korea; ^e^Department of Materials and Life Science Research Division, Korea Institute of Science and Technology, Seoul 02792, Republic of Korea*

*Corresponding author at: Department of Materials and Life Science Research Division, Korea Institute of Science and Technology, Seoul 02792, Republic of Korea; Department of Smart Green Technology Engineering, Pukyong National University, Busan 48513, Republic of Korea

*E-mail: nanostructures@kist.re.kr (Min-Seok Kim), yhkim113@pknu.ac.kr (Yong Hyun Kim)

**Figure S1.** Transmittance of natural rubber and glass substrates.


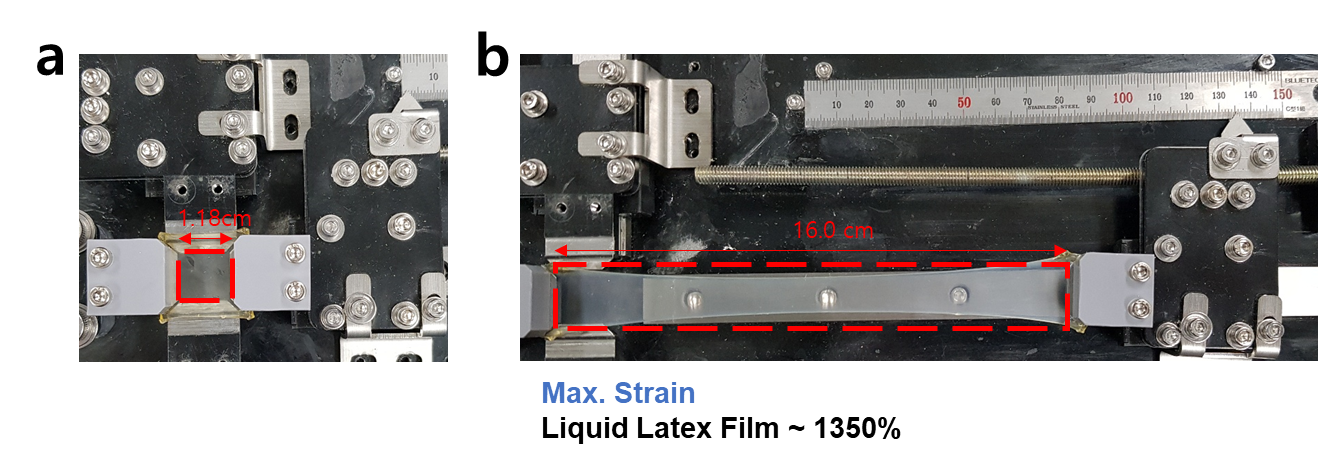


**Figure S2.** Photographs of the natural rubber/AgNW PEDOT:PSS composite film (a) at the initial length and (b) with 1350% stretching.


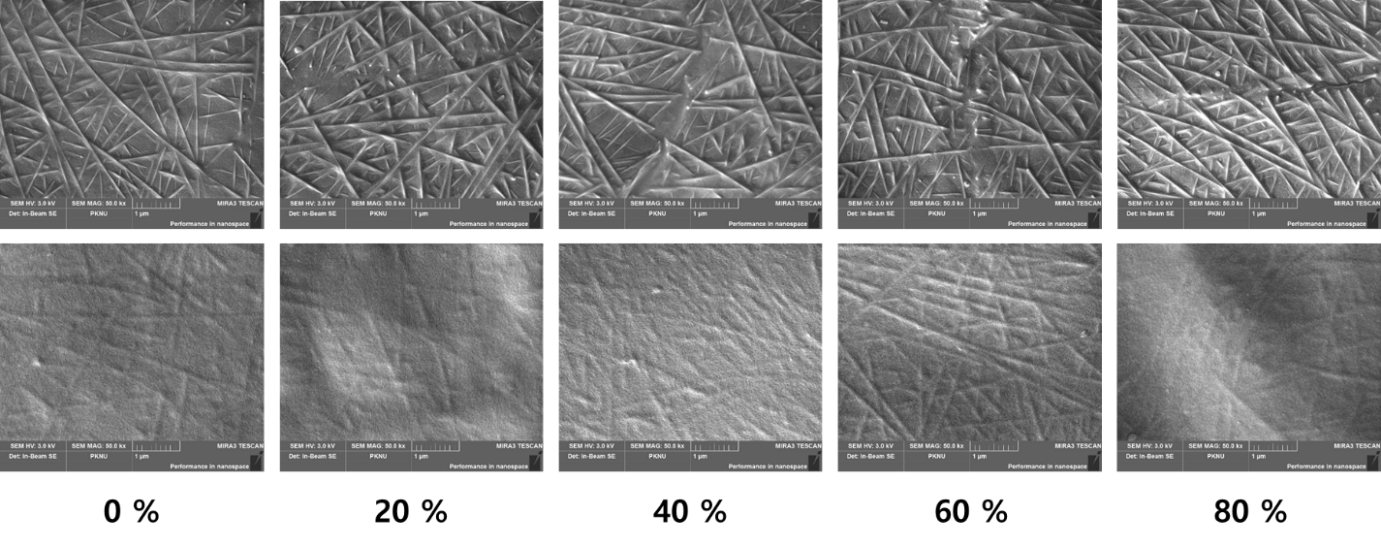


**Figure S3**. SEM images of the natural rubber/AgNW (top) and the natural rubber/AgNW PEDOT:PSS (bottom) composite films under various tensile strains. For achieving high sensing performance, we utilized the PEDOT:PSS layer (3000 rpm) with a thickness of 23 nm into the composite films, showing a higher conductivity, sensitivity, and stretchability compared to the other PEDOT:PSS films (3000 and 1500 rpm). Moreover, the PEDOT:PSS layer (3000 rpm) results in uniform coating properties.


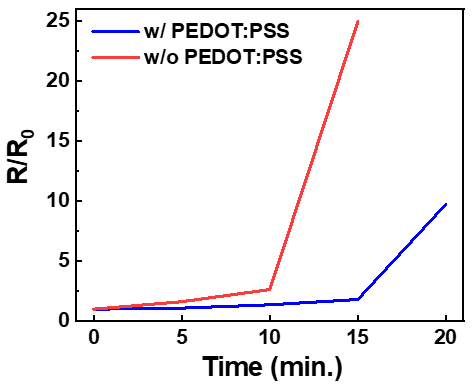


**Figure S4**. Relative resistance changes of the composite films with and without PEDOT:PSS layers which is dipped in water. The enhanced stability is also observed for the films dipped in water, which is attributed to the protection effect of the overcoated PEDOT:PSS layer.


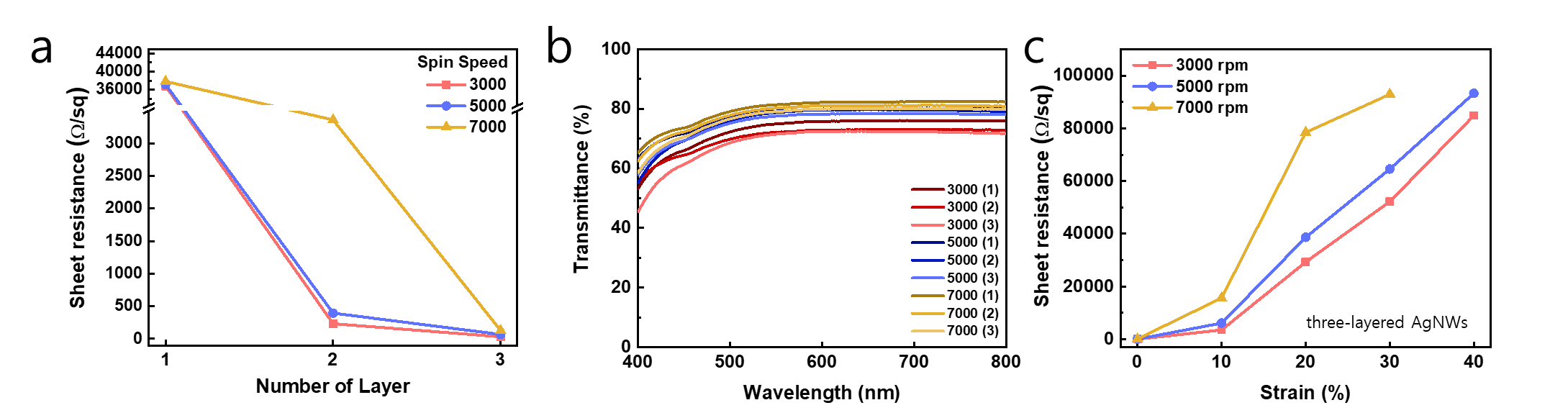


**Figure S5**. (a) Sheet resistance and (b) transmittance of the AgNW films prepared with various spin-speeds and the number of layers. (c) Changes of sheet resistance of the three-layerd AgNW films as a function of tensile strains.


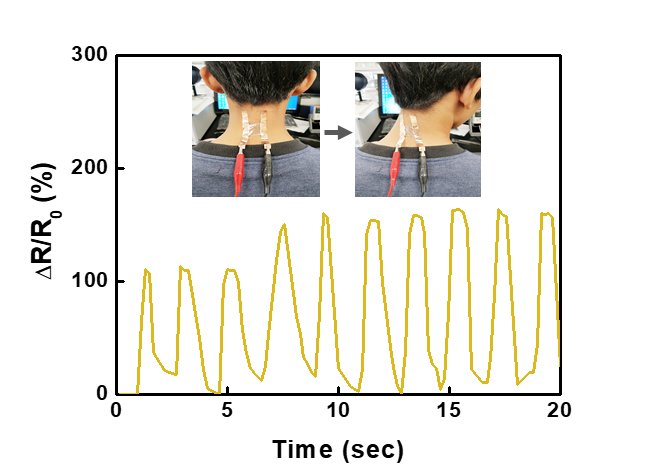


**Figure S6.** Real-time relative resistance changes of the sensors attached on the back of the neck. The sensor monitors the signal for repetitive head turning.

| **Stretchable Films** | **Elongation Break (%)** | **References** |
| --- | --- | --- |
| PDMS | 700 | *Macromolecular Rapid Communications, 38, 1700110, 2017* |
| PVA | 437 | *Carbohydrate Polymers, 184, 453, 2018* |
| PVA - NC | 518 | *Carbohydrate Polymers, 184, 453, 2018* |
| Polyurethane | 600 | *IEEE Electron Device Letters, 32, 1424, 2011* |
| cellulose | 233 | *Carbohydrate polymers 201, 446, 2018* |
| Natural rubber | 1350 | *this work* |

**Table S1**. Summary of the mechanical properties in stretchable films.

| **Material** | **Preparation** | **Characteristic** | **Source** |
| --- | --- | --- | --- |
| Natural rubber | - | Consist of 60% water, 35% cis polyisoprene and 5% non-isoprenes molecules (proteins, lipids, carbohydrates, mineral etc), highly hydrophobic, robust and stretchhable | SFX Korea |
| 11-aminoundecanoic acid | 11-AA powder (4 mg) diluted in deionized water (2 ml) and ethyl alcohol (8 ml) | The 11-AA layer consists of amine and carboxyl groups which enables linking the substrate and PVP of silver nanowires and increasing the surface energy of the natural rubber substrate. | Sigma Aldrich |
| Silver nanowire | - | AgNWs are dispersed in IPA. The nanowires are surrounded by thin PVP shells (Diameter ~21 nm, length ~22 µm). | Flexio Co., Ltd |
| PEDOT:PSS | PEDOT:PSS (10 ml) mixed with ethylene glycol (0.6 ml) and FS-31 surfactant (10 µL) | Ethylene glycol, a conductivity enhancer, greatly improves the conductivity. FS-31 non-ionic fluorosurfactnat enhances the wetting property of PEDOT:PSS. | PEDOT:PSS from Heraeus  FS-31 from Dupont |

**Table S2**. Detailed information of materials in the experiment.
